# Quantum Szilard engine for the fractional power-law potentials

**DOI:** 10.1038/s41598-020-80639-w

**Published:** 2021-01-15

**Authors:** Ekrem Aydiner

**Affiliations:** Department of Physics, Faculty of Science, İstanbul University, 34134 Istanbul, Turkey

**Keywords:** Information theory and computation, Quantum physics, Statistical physics, thermodynamics and nonlinear dynamics, Biotechnology, Medical research, Molecular medicine, Engineering, Nanoscience and technology, Physics

## Abstract

In this study, we consider the quantum Szilárd engine with a single particle under the fractional power-law potential. We suggest that such kind of the Szilárd engine works a Stirling-like cycle. We obtain energy eigenvalues and canonical partition functions for the degenerate and non-degenerate cases in this cycle process. By using these quantities we numerically compute work and efficiency for this thermodynamic cycle for various power-law potentials with integer and non-integer exponents. We show that the presented simple engine also yields positive work and efficiency. We discuss the importance of fractional dynamics in physics and finally, we conclude that fractional calculus should be included in the fields of quantum information and thermodynamics.

## Introduction

Boltzmann firstly formulated the entropy expression in the framework of Statistical Mechanics between 1872 and 1875. This definition of entropy is known as the second law of thermodynamics. However, the debates on whether the second law would be violated continued for a while. For example, in 1867 Maxwell^1^ had proposed a thought experiment in which the second entropy might be violated. So that the debate that started with Maxwell lasted almost a century^2–5^.

To test this law, Maxwell, in his thought experiment, constructs A and B boxes that are completely isolated from each other except the door between them. He suggests that both of these boxes are filled with the same type of gas and both boxes have the same temperature. According to the second law of thermodynamics, there will be no heat flow from A to B or from B to A since both boxes are in thermal equilibrium. Maxwell imagines a clever demon standing by the door and this demon can observe gas molecules on both sides. This clever demon watches all the molecules in boxes A and B. When he sees a molecule coming from box A towards the door at a higher than average speed, he opens the door and allows it to pass into box B. Similarly, it allows molecules to move at a lower than average speed in box B to pass into box A one by one. Thus, with the help of the clever demon’s control, the average velocity of molecules inside box B increases while the average velocity of those in box A decreases. Since the average kinetic energy of the particles will be interpreted as an expression of temperature, at the end of this process, this means that the temperature of box B increases, conversely, the temperature of box A decreases. If this thought experiment is possible, a heat transfer from cold to hot would be possible and this indicates that the second law can be violated.

The possible error in this thought experiment was first demonstrated by Szilárd in 1929^3^. According to Szilárd, Maxwell’s demon must generate entropy when observing the velocities of molecules, storing and comparing velocity information, and opening and closing the door. If the entropy produced by the clever demon is not taken into account, the entropy of the system may appear to have decreased. However, considering the entropy that the clever demon generates while doing the work, the total entropy is expected to increase. Szilárd analysed the demon problem of Maxwell by using a classical engine. He considered a closed box with a single particle instead of many particles. In the middle of this box, there is a compartment that will serve as a piston. This compartment divides the box into two parts. According to this setup, the particle can be located in the left or right box. If the particle is in the left box, it can push the piston to the right, and if it is in the right box, it can push the box to the left. Thus, the volume of the box containing the particle expands. Hence, work is obtained from this thermodynamic process. In this thermodynamic process, the system produces $k_{B}T$ ln 2 amount of work, where $k_{B}$ is the Boltzmann constant and *T* is the temperature of the considered system. Thus, Szilárd shows that the second law of thermodynamics is not violated. However, measuring the position of the particle causes a decrease in entropy of magnitude $k_{B}T \ln 2$.

In 1961, Landauer solved this problem^4^. He proposed an erase principle to explain the increase of the entropy. According to this principle, erasure or reset of the demon’s memory requires the minimum energy cost of at least $k_{B}T \ln 2$ in the measurement process. This ensures that the entropy of the system does not decrease for each measurement and that the total entropy of the system is increased^4,5^. Landauer’s principle has been experimentally provided using a single colloidal particle^6^. Furthermore, it was shown that the needed work to erase the memory is near $k_{B}T \ln 2$ which has been experimentally extracted from one bit of information, using a single electron engine^7^. Apart from this study, it has been shown in many experimental realizations that the Szilard engine will produce positive work^7–12^. As a result, the model proposed by Szilard played an important role in establishing a relationship between information theory and thermodynamics by through Landauer’s principle.

The quantum version of the Szilárd engine was proposed by Kim et al.^13^. Unlike the classical Szilárd engine, in the quantum version, a thin barrier is inserted and removed in the middle of the box in the thermodynamic cycle. It has been shown in various studies that the thermodynamic process realized in this way will produce positive work^14–19^. Recently, a similar version of the quantum engine has been studied by Thomas et al.^20^ for the infinite well potential. They show that the work and efficiency of this quantum Szilárd engine are positive for single and many particles. This model can be applied to any other potential where the insertion of the barrier leads to degenerate eigenstates. In the present study, we consider this model with a fractional power-law like potential. So far, the quantum engine has never been studied for fractional potentials. Based on this motivation, we discuss the work extraction and efficiency of the quantum Szilard engine which has the Stirling-like cycle under fractional power-law potential. The power-law like a fractional potential can be given in a simple form as

1 $$\begin{aligned} V(x,k) = \alpha | x \big |^{k} \end{aligned}$$

where *k* denotes the power-law exponent, and $\alpha$ is a parameter that depends on the frequency of the system. Here we note that the exponent *k* can take integer or non-integer values. For the integer value of *k*, we get well-known standard solutions. However, the solution will deviate from the standard one for the non-integer values since it leads to fractional physics. In this study, we will give some results of the fractional version of the Szilárd engine which has Stirling like a thermodynamic cycle and will discuss the importance of the fractional calculus in the quantum thermodynamics and quantum information fields.

This paper is organized as follows: In “The model and its results”, we introduce the model and give its results. In “Discussion”, we discuss the importance of the results and fractional physics in the fields of the quantum information and thermodynamics. Finally, in “Methods”, we describe give the method to obtain the numerical results of work and efficiency for this engine under fractional power-law potentials.

## The model and its results

### The Szilard engine under fractional power-law potential

The quantum Szilard engine which has the Stirling cycle^21–24^ under the infinite quantum well potential^20^ was introduced in the introduction section. Here, in this study, we will carry out the work efficiency of this engine for a single quantum spinless particle under the fractional power-law potential given in Eq. (1).

**Figure 1:**
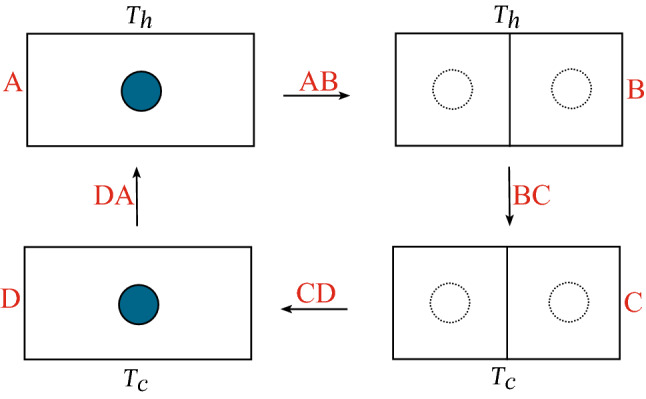
The four stages of the Stirling cycle. In A and D, solid circles represent the particles. Dashed circles in B and C represent the uncertainty in the position of the particle. Box A and B contacts the hot bath temperature $T_{h}$, however, the boxes C and D contact the cold bath temperature $T_{c}$. The stages BC and DA are isochoric processes. Stage AB is the isothermal insertion process, while stage CD is the isothermal removal process.

The schematic picture of the thermodynamic cycle processes to work extracted is given in the Fig. 1. According to the Stirling engine schema, AB and CD are the isothermal processes while BC and DA represent isochoric processes. All thermodynamic steps are given as follows: The cyclic process starts with A. In the A stage, the particle which is under a single power-law potential in box A interacts with a heat bath with a higher temperature of $T_{h}$. In the AB stage, a thin potential barrier is completely inserted in the box at the same temperature. During the quasi-static insertion process, the system is in equilibrium with a hot bath temperature of $T_{h}$. After this isothermal process, the box B behaves like the two double power-law potentials. However, it is assumed that in the BC stage the system is contacted to a heat bath with a lower temperature of $T_{c}$. In this process, the system undergoes the isochoric temperature exchange. To continue the thermodynamic cycle, the inserted thin barrier is isothermally removed in the CD stage. The system is in equilibrium with cold bath temperature $T_{c}$ during the quasi-static removal process. After the CD process, the particle is again under a single power-law potential in box C with a hot bath temperature of $T_{c}$. In the final DA stage, the system is contacted to a heat bath with a higher temperature of $T_{h}$ and is completed cycle. In the last stage, the system also undergoes the isochoric temperature exchange.

The work to be extracted from this thermodynamic cycle is given by

2 $$\begin{aligned} W(k) = k_{B} T_{h} \ln \frac{Z_{B}(k)}{Z_{A}(k)} - k_{B} T_{c} \ln \frac{Z_{C}(k)}{Z_{D}(k)} \end{aligned}$$

where $k_{B}$ is the Boltzmann constant, $T_{h}$ and $T_{c}$ indicate the hot and cold temperatures, and $Z_{i}$ ($i=A,B,C,D$) are the canonical partition functions of the system. In a similar way, the efficiency of the cycle is given by

3 $$\begin{aligned} \eta (k) = \frac{\text {total work done}}{\text {heat supplied}} = 1 + \frac{Q_{BC} + Q_{CD} }{Q_{DA} + Q_{AB} } \end{aligned}$$

where $Q_{j}$ (*j*=BC, CD, DA, AB) are the heat exchanges for the stages. The quantities in the work *W* in Eq. (2) and efficiency $\eta$ in Eq. (3) will be obtained taking into account the thermodynamic stages mentioned above. In the calculation, for simplicity we neglect some extra energy cost for instance needed energy for insertion and removal of the barrier and needed energy for coupling or decoupling of the system to the heat baths. The details of the calculation of work *W* in Eq. (2) and efficiency in Eq. (3) will be discussed in Method Section.

### Numerical results

**Figure 2:**
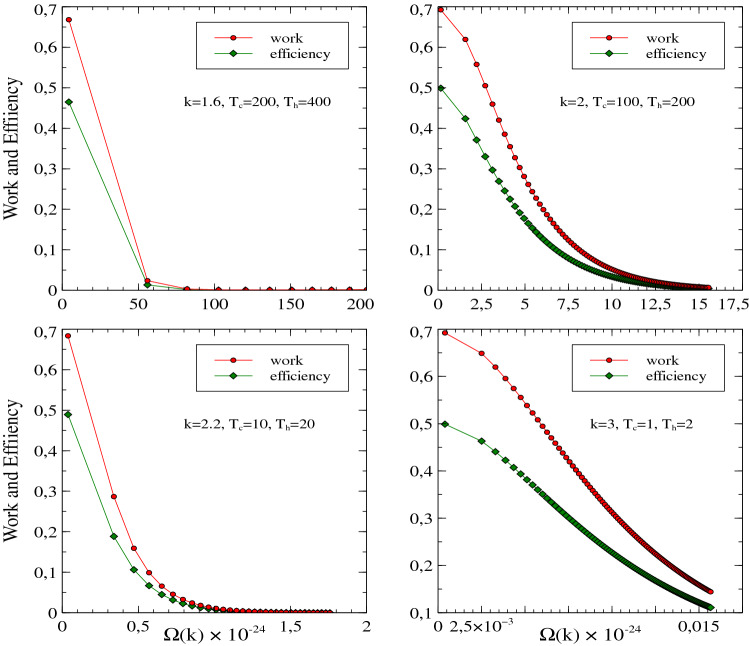
Work and efficiency versus $\Omega (k)$ for several *k* and temperature values. Here, we have set the constants as $m=19.11\times 10^{-11}$, $k_{B}=1.380649\times 10^{-23}$ and $h=6.62607015\times 10^{-34}$.

In the presented model, we compute energy eigenvalues corresponding to the potential with help of WKB approximation. By using these eigenvalues we numerically computed the work *W* in Eq. (2) and efficiency $\eta$ in Eq. (3) of a Stirling-like cycle presented in Fig. 1 for various *k* values. Obtained numerical results are given in Fig.2. Both thermodynamic quantities in Fig.2 are plotted as a function of the parameter $\Omega (k)$ which is defined in Eq. (7). The parameter $\Omega (k)$ is a function of the frequency $\omega$. For instance, this parameter for $k=2$ and $\alpha =\frac{1}{2}m\omega ^{2}$ reduce to $\hbar \omega$. Fig.2 clearly shows the frequency dependence of the work *W* and efficiency $\eta$ for various *k* values. Here, we have set the constants as $m=19.11\times 10^{-11}$, $k_{B}=1.380649\times 10^{-23}$, and $h=6.62607015\times 10^{-34}$.

As it can be seen from Fig.2 the model under power-law potential with integer and non-integer exponents yields positive work and efficiency for a single particle. Firstly, we note that the numerical procedure requires more attention since the obtained results are tightly dependant on the parameters *k*, $T_{h}$ and $T_{c}$. We see that the choice of these parameters affects the result dramatically. Therefore, to find meaningful results the parameter intervals must be carefully scanned.

We present the results for various *k* and heat bath temperatures in Fig.2. One can see from the figure that work and efficiency are positive and maximum for very low-frequency values although the parameters are chosen differently. However, the values of *W* and $\eta$ slowly decrease when the frequency parameter $\Omega (k)$ increases. The maxima values of the work and efficiency for different power-law exponents and temperature values are compatible with the results of Ref.^20^ where these quantities have been obtained as a function of the box size *a* which indicates the position of the inserted barrier. In our work, the position of the inserted barrier is fixed in the B or C boxes. We obtain work and efficiency depending on particle frequency for a fractional power-law potential.

As a result, in this study, we show that it is possible to compute quantities for the non-integer *k* values which denotes fractional potential which can be created by deformed fields and fractal space or porous structures. This simple model can be regarded as a toy model to discuss the quantum Szilard engine with single-particle under fractional power-law potential. Here, we try to generalize the well known model in Ref.^20^ to fractional power-law potential. We have not considered the limit cases such as the low-temperature limit. We also limited the work to a single particle for simplicity. However, the model and results can be generalized for many particle quantum systems.

## Discussion

The classical and quantum version of the Szilard engine has been studied in detail theoretically and experimentally^7–20^. In these studies, it is shown that the second law of thermodynamics will not actually be violated, if a more complete analysis is made of the whole system including the demon. On the other hand, it is clearly understood by these studies that there is a connection between thermodynamic entropy and information entropy which leads positive work. It has been understood that it is possible to produce quantum heat engines in nano-size. Nowadays, the interest in these machines is increasing due to their potential technological applications.

In our study, we show that the quantum Szilard engine under fractional potential also yields positive work as a substance working. The toy model presented here is not new. However, unlike the others, this is the first time that fractional potential has been included in the quantum thermodynamic discussions. In this sense, this study may play an important role in bringing attention to the fractional quantum engine.

As far as we know heat engines and information theory have not been studied using fractional calculation methods in the literature. However, fractional theory can be included in these areas. We mention that the integer-based model and Hamiltonian solutions are idealized models and solutions that completely fit to the Euclidean description. Whereas the fractional calculus includes the Euclidean-based Newtonian calculus area which is based on the local point property of the object. Fractional calculus is characterized by fractional derivative and fractional integral^25^. When we think of it as a mathematical domain, fractional calculus covers the Newtonian calculus. Many events and processes such as non-local, distributed, non-Markovian, non-Newtonian, and non-differentiated fields and dynamics can cause disordered structure and complexity. Therefore, these processes can be modelled with the help of the fractional calculus^25–31^. Indeed, the fractional dynamics can be seen in the many physical systems such as dielectrics, spin glasses, proteins, anomalous diffusion, anomalous wave propagation, biological materials etc^25–31^. All of these imply that the fractional calculus should be included in the fields of quantum information and thermodynamics.

Here, we obtain the work and efficiency of the model without using fractional calculus tools. But, by applying fractional calculus one can discuss thermodynamic problems, quantum heat engines, or refrigerators. Because we know that if there are non-local, distributed, non-Markovian, non-Newtonian dynamics in any physical system, these effects will appear on the adiabatic processes, probabilities, and eigenvalues of the system in the thermodynamic cycle. Therefore, it is important to incorporate fractional dynamics into quantum information and quantum thermodynamic systems. In this study, we will be content with stating that this issue will gain importance in the future. Hence, it would not be wrong to predict that the concept of fractional heat engines will also enter the literature.

On the other hand, today, we know that both quantum engine and quantum Szilard engine were experimentally realized^7–12,32–38^. Therefore, we state that based on previous experimental methods and by implementing, for instance, fractal geometries or structures with porous surfaces to the experimental setup, it may possible to realize a fractional quantum heat engine in practice.

## Methods

### Energy eigenvalues for the fractional power-law potential

To carry out the work and efficiency corresponding to the thermodynamic cycle of the quantum Szilard engine we will use the canonical partition function and heat exchange for each stage. Therefore, to construct partition function and to obtain other thermodynamic quantities for instance internal energy and heat for the cycle we need energy eigenvalues of the particle systems for the power-law potential given in Eq. (1). For the present case, energy eigenvalues can be obtained from the solution of the Hamiltonian of the system:

4 $$\begin{aligned} H(x,k) = \frac{{\hat{p}}^{2}}{2m} + {\hat{V}}(x,k). \end{aligned}$$

For integer values i.e., $k=1,2,3,4$, it is very easy to obtain an analytical solution of any quantum mechanical system with a single particle. However, for the non-integer *k* values it is not possible to obtain exact results. On the other hand, the non-integer *k* values indicate the fractional potential which requires using fractional calculus or other approximation methods. One of the simple methods is the WKB approximation. Energy eigenvalues of

5 $$\begin{aligned} H(x,k)|n> = E_{n, k} |n>, \quad n = 0,1,2,... \end{aligned}$$

can be obtained in a simple form as

6 $$\begin{aligned} E_{n,k} = \Omega (k) \left( n + \frac{\gamma }{4}\right) ^{2k/(k+2)} \end{aligned}$$

where $\Omega (k)$ depends on the frequency *w* of the system, and is given by

7 $$\begin{aligned} \Omega (k) = \alpha \left[ \frac{\hbar \sqrt{\pi }}{\sqrt{2 m \alpha }} \frac{\Gamma \left( \frac{1}{k} + \frac{3}{2}\right) }{\Gamma \left( \frac{1}{k} + 1\right) }\right] ^{2k/(k+2)} \end{aligned}$$

where $\gamma$ denotes the Maslov index which represent a correction to the quantum number. The final result in Eq. (6) can also be found in Griffiths posed as a problem^39^. The index $\gamma$ takes different values. For instance, $\gamma = 2$ the harmonic oscillator , $\gamma = 3$ for the triangular-well potential, and $\gamma = 4$ corresponds to the infinite square well. We note that for $k=2$, $\alpha =\frac{1}{2}m\omega ^{2}$ and $\gamma =2$, from Eq. (6) we get energy of the harmonic oscillator

8 $$\begin{aligned} E_{n} = \left( n + \frac{1}{2}\right) \hbar \omega . \end{aligned}$$

On the other hand, $k < 2$ expresses loosely binding potentials that leads to the spacing between energy levels decreasing with increasing quantum number *n* and $k>2$ corresponds to the tightly binding potentials which exhibit increasing energy levels with increasing quantum number *n*.

### Work and efficiency

Now we discuss the work and efficiency for this engine model following the method given in Ref.^20^. To compute work *W* in Eq. (2) and efficiency in Eq. (3) we need to know partition functions, internal energies, and heat exchanges for all processes.

To compute the work *W* in Eq. (2) we have to construct partition functions $Z_{A}$, $Z_{B}$, $Z_{C}$ and $Z_{D}$. Therefore, we will give all of them subsequently below. Firstly, we assume that the system is connected to a heat bath with a higher temperature $T_{h}$. The partition function $Z_{A}(k)$ for the high temperature $T_{h}$ is simply given as

9 $$\begin{aligned} Z_{A}(k) = \sum _{n=1}^{\infty } \exp [-E^{A}_{n,k}/k_{B}T_{h} ] \end{aligned}$$

where $E^{A}_{n,k}$ is the energy eigenvalue of the particle in the box A. By using Eq. (6), $E^{A}_{n,k}$ is written as

10 $$\begin{aligned} E^{A}_{n,k} =\Omega (k) \left( n + \frac{\gamma }{4}\right) ^{2k/(k+2)} \end{aligned}$$

which has non-degenerate form. If Eq. (10) is inserted into Eq. (9), the partition function $Z_{A}(k)$ can be written as

11 $$\begin{aligned} Z_{A}(k) = \sum _{n=1}^{\infty } \exp \left[ \frac{ \Omega (k)}{k_{B}T_{h}} \left( n + \frac{\gamma }{4}\right) ^{2k/(k+2)} \right] . \end{aligned}$$

The energy eigenvalue and the partition function for box B will be different from box A. Because, we assume that, in the AB stage depicted in Fig. 1, a very thin potential wall is isothermally inserted slowly in the middle of the box at the same temperature. When the wall is completely inserted in the box, the energy levels will be degenerate as explained in Ref.^20^. In this case, for box B, energy eigenvalue is given by

12 $$\begin{aligned} E^{B}_{n,k} = \Omega (k) \left( 2n + \frac{\gamma }{4}\right) ^{2k/(k+2)} \end{aligned}$$

Hence, the corresponding partition function is given by

13 $$\begin{aligned} Z_{B}(k) = \sum _{n=1}^{\infty } 2 \exp \left[ \frac{ \Omega (k)}{k_{B}T_{h}} \left( 2n + \frac{\gamma }{4}\right) ^{2k/(k+2)} \right] \end{aligned}$$

where the pre-factor 2 in Eq. (13) is written since the boxes are divided into two by the barrier which leads to energy levels two-fold degenerate.

In the BC stage, we assume that the system leaves the heat bath with higher temperature $T_{h}$ and contacts the other heat bath with lower temperature $T_{c}$. Therefore, we compute the partition function for C and D in the case of the low temperature $T_{c}$. The energy eigenvalue of box C is

14 $$\begin{aligned} E^{C}_{n,k} = \Omega (k) \left( 2n + \frac{\gamma }{4}\right) ^{2k/(k+2)} \end{aligned}$$

which has also degenerate energy levels like box B. The partition function for box C is given by

15 $$\begin{aligned} Z_{C}(k) = \sum _{n=1}^{\infty } 2 \exp \left[ \frac{ \Omega (k)}{k_{B}T_{c}} \left( 2n + \frac{\gamma }{4}\right) ^{2k/(k+2)} \right] . \end{aligned}$$

Finally, we can write the energy eigenvalue of the box D as

16 $$\begin{aligned} E^{D}_{n,k} = \Omega (k) \left( n + \frac{\gamma }{4}\right) ^{2k/(k+2)} \end{aligned}$$

and the partition function is given by

17 $$\begin{aligned} Z_{D}(k) = \sum _{n=1}^{\infty } \exp \left[ \frac{ \Omega (k)}{k_{B}T_{c}} \left( n + \frac{\gamma }{4}\right) ^{2k/(k+2)} \right] . \end{aligned}$$

Finally, in the last stage DA, the system leaves the heat bath with low temperature and contacts again higher temperature one. As a result, the work *W* in Eq. (2) for this Striling-like cycle can be numerically computed with help of Eqs. (11), (13), (15) and (17).

On the other hand, to obtain the efficiency $\eta$ in Eq. (3) we have to construct the heat exchanges $Q_{AB}$, $Q_{BC}$, $Q_{CD}$ and $Q_{DA}$. For this isothermal process AB, the heat exchanged $Q_{AB}$ can be obtained as

18 $$\begin{aligned} Q_{AB} = U_{B} - U_{A} + k_{B} T_{h} \ln \frac{Z_{B}(k)}{Z_{A}(k)} \end{aligned}$$

where $U_{A}$ and $U_{B}$ are the internal energies of the box A and B, respectively, which can be obtained from

19 $$\begin{aligned} U_{A,B} = - \frac{\partial Z_{A,B}(k)}{\partial \beta _{h}} \end{aligned}$$

where $\beta _{h}=1/k_{B}T_{h}$. Another isothermally stage CD leads to heat exchange $Q_{CD}$ which is written as

20 $$\begin{aligned} Q_{CD} = U_{D} - U_{C} + k_{B} T_{c} \ln \frac{Z_{D}(k)}{Z_{C}(k)} \end{aligned}$$

where $U_{C}$ and $U_{D}$ are internal energies for box C and D, respectively which can be found from

21 $$\begin{aligned} U_{C,D} = - \frac{\partial Z_{C,D}(k)}{\partial \beta _{c}} \end{aligned}$$

where $\beta _{c}=1/k_{B}T_{c}$ is the inverse temperature.

Now, we can focus on the heat exchange for two isochoric processes BC and DA. The amount of exchanged heat $Q_{BC}$ during isochoric process BC is given by

22 $$\begin{aligned} Q_{BC} = U_{C} - U_{B} \end{aligned}$$

At this stage the system is connected to the lower temperature bath at $T_{c}$. On the other hand, the heat exchanged $Q_{DA}$ for another isochoric process DA is given by

23 $$\begin{aligned} Q_{DA} = U_{A} - U_{D}. \end{aligned}$$

At the final stage, the system is again connected to the higher temperature bath at $T_{h}$. As a result, by using Eqs. (18), (20), (22) and (23), the efficiency $\eta$ in Eq. (3) can be numerically obtained.
